# Social Media Mining of Long-COVID Self-Medication Reported by Reddit Users: Feasibility Study to Support Drug Repurposing

**DOI:** 10.2196/39582

**Published:** 2022-10-03

**Authors:** Jonathan Koss, Sabine Bohnet-Joschko

**Affiliations:** 1 Department of Management and Entrepreneurship Faculty of Management, Economics and Society Witten/Herdecke University Witten Germany

**Keywords:** social media mining, drug repurposing, long-COVID, crowdsourcing, COVID-19, Reddit, social media, content analysis, network analysis, recognition algorithm, treatment

## Abstract

**Background:**

Since the beginning of the COVID-19 pandemic, over 480 million people have been infected and more than 6 million people have died from COVID-19 worldwide. In some patients with acute COVID-19, symptoms manifest over a longer period, which is also called “long-COVID.” Unmet medical needs related to long-COVID are high, since there are no treatments approved. Patients experiment with various medications and supplements hoping to alleviate their suffering. They often share their experiences on social media.

**Objective:**

The aim of this study was to explore the feasibility of social media mining methods to extract important compounds from the perspective of patients. The goal is to provide an overview of different medication strategies and important agents mentioned in Reddit users’ self-reports to support hypothesis generation for drug repurposing, by incorporating patients’ experiences.

**Methods:**

We used named-entity recognition to extract substances representing medications or supplements used to treat long-COVID from almost 70,000 posts on the “/r/covidlonghaulers” subreddit. We analyzed substances by frequency, co-occurrences, and network analysis to identify important substances and substance clusters.

**Results:**

The named-entity recognition algorithm achieved an F1 score of 0.67. A total of 28,447 substance entities and 5789 word co-occurrence pairs were extracted. “Histamine antagonists,” “famotidine,” “magnesium,” “vitamins,” and “steroids” were the most frequently mentioned substances. Network analysis revealed three clusters of substances, indicating certain medication patterns.

**Conclusions:**

This feasibility study indicates that network analysis can be used to characterize the medication strategies discussed in social media. Comparison with existing literature shows that this approach identifies substances that are promising candidates for drug repurposing, such as antihistamines, steroids, or antidepressants. In the context of a pandemic, the proposed method could be used to support drug repurposing hypothesis development by prioritizing substances that are important to users.

## Introduction

### Background

Since the beginning of the COVID-19 pandemic, over 480 million people have been infected and more than 6 million people have died from COVID-19 worldwide [1]. In some patients with acute COVID-19, symptoms manifest over a longer period of time [2]. Owing to this phenomenon, the term “long-COVID” (LC) has emerged [3]. LC refers to both postacute (lasting longer than 4 weeks) and chronic (lasting longer than 12 weeks) symptoms [3,4]. At least one symptom persists in 32%-87% of previously hospitalized patients [4]. Furthermore, the incidence of LC is estimated to range between 10% and 35% in individuals who have not been hospitalized [5]. The economic costs associated with LC symptomatology could be significant. Reynolds et al [6] stated that chronic fatigue syndrome, which has similar characteristics to LC [7], leads to a 37% reduction in household productivity and a 54% reduction in labor force productivity in the United States. Unmet medical needs have motivated immense research activities. ClinicalTrials.gov lists more than 7000 studies in the field of COVID-19, including more than 600 LC-specific studies [8].

### Retrospective Clinical Analysis

The large number of ongoing studies highlights a key challenge in drug development. There are numerous substances that are potentially effective. It is therefore essential to identify promising substances and narrow down the number of potential drug candidates to those showing the most promise. Given the urgency, scarcity of financial resources, and the high risk of failure in pharmaceutical research [9], drug repurposing (DR) appears to be a promising strategy for LC drug development. The exploitation of existing drugs for new therapeutic purposes usually leads to shorter development cycles with lower costs [10]. For example, existing drugs have proven to be safe for use in humans. Accordingly, phase I clinical trials are not required [10]. From a historical perspective, DR has often been serendipitous [10,11], but systematic approaches also exist to identify promising target leads [12]. One of these approaches is retrospective clinical analysis [12], which has already been used in the context of the COVID-19 pandemic [12]. Retrospective clinical analysis involves learning from real-world experience (eg, evaluating clinical case reports) to hypothesize applications of existing drugs for new indications [13].

### Mining Patients’ Experiences From Social Media

Traditionally, retrospective clinical analysis is based on information that is collected and stored in databases, explicitly dedicated for health care system–related applications. Signals for potential DR are subsequently generated by professionals analyzing the data. Nowadays, there is a growing awareness in medical research that the collective intelligence of the affected patients, jointly searching for a solution to improve a medical condition, can be a driver of innovation [14,15] that is leveraged in the innovation process known as “crowdsourcing” [14,16]. While traditional crowdsourcing aims at active collaboration (eg, between a pharmaceutical company and an external patient group), online forums enable a passive approach to collecting real-world data by offering relevant content for analysis, which is also called “passive crowdsourcing” [17,18]. For instance, researchers have analyzed data from disease-specific social media platforms to identify medications that are used outside the approved indication (off-label use) [10,19]. Off-label use provides information to support hypotheses regarding DR [10,19]. These approaches save time and costs associated with data collection, while incorporating patients’ real-world experiences. However, social media mining (SMM) [19], a term that refers to the collection of methods used for conducting passive crowdsourcing, poses significant risks in terms of bias [10,19]. For instance, owing to the age-related user behavior on social media platforms, the data might not be generalizable to the whole population [10].

### Generating Hypotheses on DR From Discussions Among Long-Haulers

In this study, we aimed to capture substances such as medications and supplements that are relevant to the coping strategies of patients with LC. Accordingly, we applied the principle of retrospective clinical analysis using passive crowdsourcing by applying SMM. Since there is no approved drug for LC, promising LC candidates based on off-label properties could not be determined. Instead, we used an exploratory method, mainly consisting of the application of named-entity recognition (NER) and network analysis, aiming to provide an overview of different treatment strategies and important compounds from the patient’s perspective for DR hypothesis generation. Methodologically similar approaches have previously been used to identify substances used in self-medication regarding opioid withdrawal [20] or to monitor potential drug interactions and reactions [21]. Furthermore, network analysis has been used to explore discussions related to certain diseases [22] or to explore the public perspective on vaccines [23,24]. For example, Lewis et al [22] used network analysis to analyze reasons for older adults to join a diabetes online community. Luo et al [24] used network analysis to explore public perceptions of the COVID-19 vaccine.

To our best knowledge, this study is the first to explore treatment strategies and important medications to support DR hypothesis generation by applying network analysis.

### Research Objectives

The aim of this feasibility study was to evaluate whether the proposed method can be employed to support DR hypothesis generation based on the experiences of affected individuals shared on Reddit. To this end, we first explored which substances are mentioned in LC online discussions regarding self-medication. Second, we investigated whether there are clusters of substances often discussed together, indicating treatment strategies. Third, we attempted to identify the most important substances in these clusters to indicate respective treatment strategies.

## Methods

### Overview

**Figure 1:**
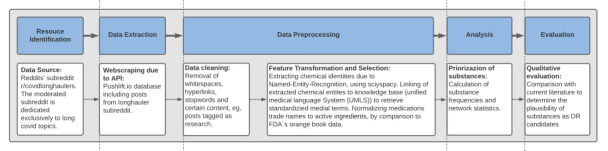
End-to-end detailed study workflow. The workflow can be divided into the following steps: resource identification, data extraction, data preprocessing, analysis, and evaluation. API: application programming interface; DR: drug repositioning; FDA: Food and Drug Administration.

The methodology used in this study consists of the following steps: (1) extraction of appropriate data, (2) detection of substance entities mentioned in users’ posts using NER, and (3) analysis of substance frequencies and co-occurrence networks of substance entities. Figure 1 outlines the end-to-end workflow in detail.

### Data Source and Extraction

Reddit is a social media platform that is organized in theme-specific forums called “subreddits” [20]. The data extraction process was performed using Pushshift [25], which is a platform that collects Reddit data and has been available to researchers since 2015 [25]. The extracted data consist of posts and metadata from the subreddit “/r/covidlonghaulers,” which has already been used to explore LC symptoms [26,27]. This subreddit is actively moderated by specific users and provides a medium for LC-related discussions. The content is subject to strict rules prohibiting the promotion of alternative treatment, misinformation, and conspiracy theories. As of January 3, 2022, the subreddit had over 24,000 subscribers and 20,000 threads. Users self-report their LC experiences such as discussing symptoms [26] and medications.

**Figure 2:**
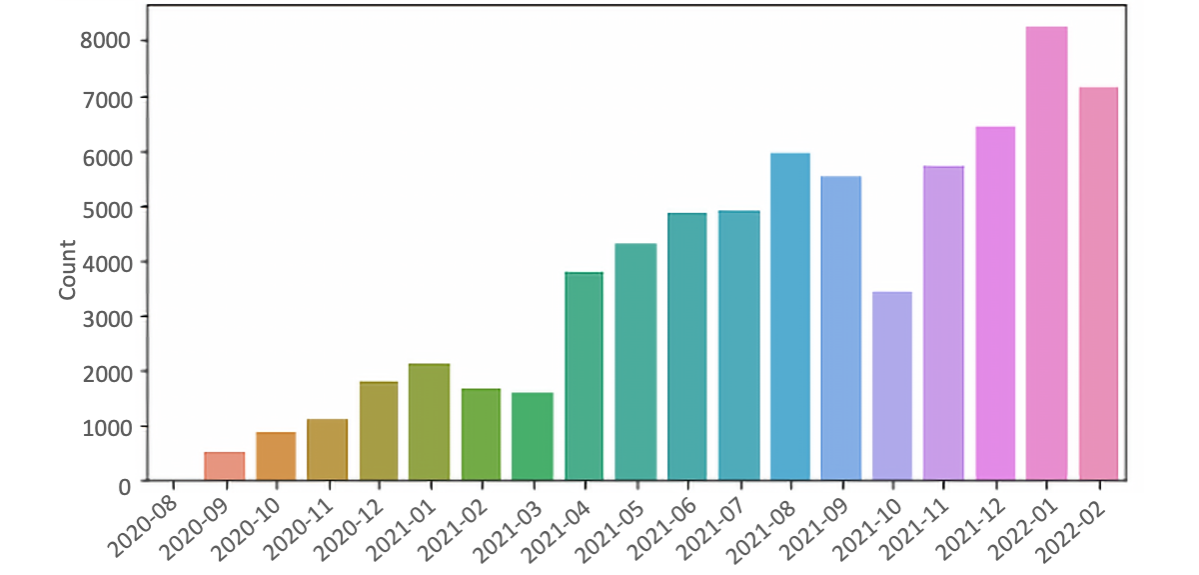
Overview of the number of posts at different dates.

Beyond the extraction of posts, metadata such as the username, date of the post, or the so-called “link flair text” can be extracted. Link flair text represent thematic tags that are used to associate posts (initial and subsequent posts) with specific categories. This provides researchers with the ability to exclude data unrelated to the analysis. For example, posts tagged as articles, research articles, or humor posts were excluded from the analysis (see Multimedia Appendix 1). Additionally, posts without tags were excluded. The analyzed data included 68,268 posts written by 8717 users between August 31, 2020, and March 1, 2022 (Figure 2).

### Substance Entity Extraction

First, the text was preprocessed to improve the data quality. For example, hyperlinks, tabs, and blank lines were removed. The substances of interest mentioned by patients in posts needed to be extracted and structured for subsequent analysis [19] using NER [28]. We defined substances of interest as explicitly mentioned substances or groups of substances that can be considered as treatments. For instance, we captured conventional supplements (eg, vitamin supplements) or prescription medications (eg, antidepressants) that were discussed by users. ScispaCy provides several NER models related to medical issues [29] and was used to extract symptoms from LC Reddit posts [27]. In principle, two ScispaCy models are applicable for this purpose. The first model is the en_ner_bc5cdr_md model [30], which can detect chemical substances and diseases. Since the module covers chemical substances in general and not specific medications or dietary supplements, we defined stop words such as “ethanol” to narrow the focus of the analysis to substances of interest. The second model is the Med7 model [31], which specifically focuses on drug extraction. Running both models together yielded the best results [27]. Negated substances were excluded from the extraction by considering negotiation terms. For this purpose, we added the Negex algorithm [32] to the NER ScispaCy pipeline. Negex identifies different forms of negation patterns and was initially developed for application to clinical texts [32]. Subsequently, the named entities were normalized and filtered to improve data quality for subsequent analyses. Therefore, the extracted entities were matched against an external knowledge base and were either replaced with standard medical vocabulary or discarded if no match was found. For this purpose, we used the ScispaCy entity linker, which matches entities with the unified medical language system (UMLS) knowledge base [33]. The EntityLinker pipeline performs a string nearest-neighbor search for entities to match them with the UMLS concepts [29]. We considered 0.85 as a threshold value for the overlap with UMLS concepts [29]. To evaluate the entity extraction performance, 500 randomly selected posts from the entire corpus were manually annotated. For data annotation, two annotators were involved. The intercoder reliability was 0.94. The F1 score was used as the evaluation metric [34]. By applying inexact string matching between data from the Food and Drug Administration’s Orange Book [35] and the extracted entities, brand names were normalized by their active ingredient [36]. For instance, “zyrtec” was replaced with “ceterizine hydrochloride.”

### Network Analysis

The network analysis method offers the possibility of visualizing and evaluating relationships in text. In this study, we used network analysis to obtain an overview of the spectrum of substances and identify potential substance clusters. Similar approaches have been used to identify substances and their effects, including self-medication in opioid withdrawal [20] or pharmacovigilance settings [21].

Features for network analysis consist of the nodes (represented by the extracted entities) and the “edges,” which represent the relationship between the nodes as weight based on the co-occurrence of entities. A co-occurrence was defined as the mention of two or more substances in one post [37,38]. Duplicates of entities within one post were removed to avoid assigning more weight to longer posts that mention specific entities more frequently. Subsequently, the information on the co-occurrence of substances was converted into a pointwise mutual information (PMI) matrix [20,39,40]. We only considered associations between entities that co-occurred more frequently than expected based on their overall frequency, also called positive PMI, which was proven to be beneficial for extracting semantic representations [20,41]. To improve the quality of the visualization and analysis, substances occurring less than 10 times and node pairs below the average PMI weight were excluded [22,42]. False-positive nodes were manually removed. Using the PMI matrix, an undirected graph was created and analyzed using Gephi software. Gephi is an open-source software for network analysis, which allows spatialization, filtering, navigation, manipulation, and clustering of entities [43].

### Community Detection

We used clustering, also referred to as “community detection,” to identify potential drug and/or supplement strategies for LC. Community detection describes the clustering of nodes (in our case, substances) that are strongly associated with each other according to their edges. Hence, a cluster consists of substances that are strongly associated and discussed with one another. Communities can be determined using various clustering algorithms. For this purpose, the relatively new Leiden algorithm was used [44]. In contrast to the Louvain algorithm, which has been widely used in network analysis in the past, the Leiden algorithm has several advantages [45] such as having more meaningful partitioning [44]. The modularity value (Q) was used as the quality function [44]. A Q value of at least 0.3 implies meaningful clustering [44]. The result of clustering, and consequently Q, was significantly determined by the preselected resolution [46]. Following an iterative process, we aimed to find a proper balance between the number and relevance of the discovered communities and the resulting modularity by applying different resolution values [46]. To analyze the most important substances in the network, we calculated the degree centrality (number of linkages of a node) [47]; the higher the centrality, the more important the substance is in the network [47,48].

## Results

### Substances in Self-Reports

The NER algorithm achieved an F1 score of 0.67 (precision=0.69, recall=0.66). Error analysis was performed on the incorrectly labeled entities. Errors were classified into lexical and dictionary errors [49]. A lexical error (38.5%) refers to the case in which users employ a variety of terms when referring to the substances they use. For example, our model failed to detect and extract the term “benzos,” since it is a slang abbreviation for the drug “benzodiazepine” and was not indexed in the model. Another example of expressions that our model did not recognize are compound terms of more than a single word (eg, “anti histamines”) with the algorithm only extracting “histamines” and was thus missing the preceding word “anti,” giving the extracted entity a different meaning.

A dictionary error (61.5%) refers to certain terms that are not specifying a concrete substance but rather a substance group; for example, “electrolytes” were not captured, whereas explicitly mentioned substances representing electrolytes such as “magnesium” were reliably detected. Furthermore, the algorithm extracted substances that are not considered as treatments, such as “chlorine.”

**Figure 3:**
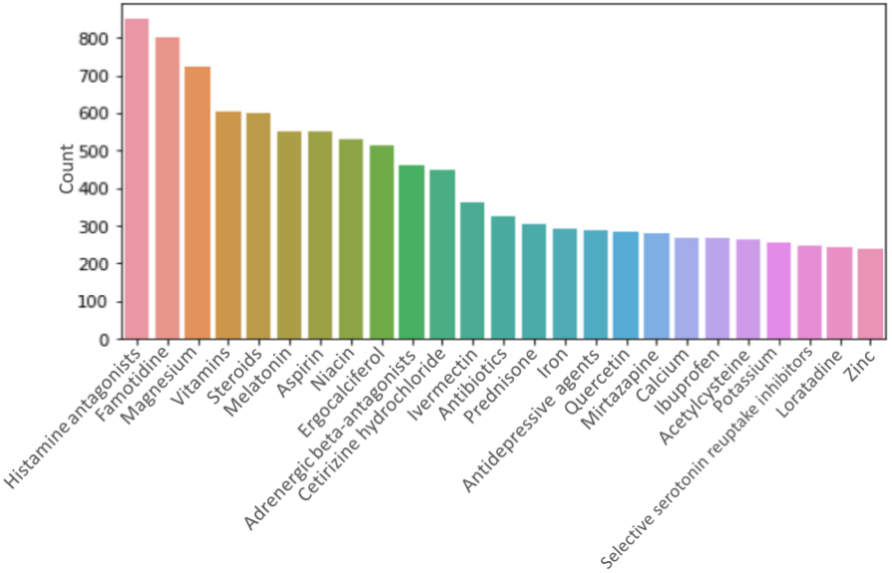
The 25 most frequently mentioned substances that appeared at least once in a post. For example, “histamine antagonists” were discussed in more than 800 different posts.

A total of 28,447 substance entities and 5789 word-co-occurrence pairs were extracted. “Histamine antagonists,” “famotidine,” “magnesium,” “vitamins,” and “steroids” were the most frequently mentioned substances that appeared at least once in a post (duplicate mentions of a substance in a post were disregarded) (Figure 3). A list of all substances can be found in Multimedia Appendix 1.

The most frequent word pairs are listed in Table 1. For example, the pairing that occurred most frequently with 218 mentions was cetirizine hydrochloride–famotidine.

**Table 1:** The most frequent co-occurrences.

Rank	Substance–Substance pair	Frequency (number of mentions)
1	Cetirizine Hydrochloride–Famotidine	218
2	Famotidine–Histamine Antagonists	135
3	Potassium–Magnesium	106
4	Famotidine–Loratadine	98
5	Ergocalciferol–Magnesium	96
6	Cetirizine Hydrochloride–Histamine Antagonists	95
7	Aspirin–Famotidine	88
8	Loratadine–Histamine Antagonists	82
9	Zinc–Ascorbic Acid	78
10	Famotidine–Melatonin	78

### Substance Clusters

#### Overview

**Figure 4:**
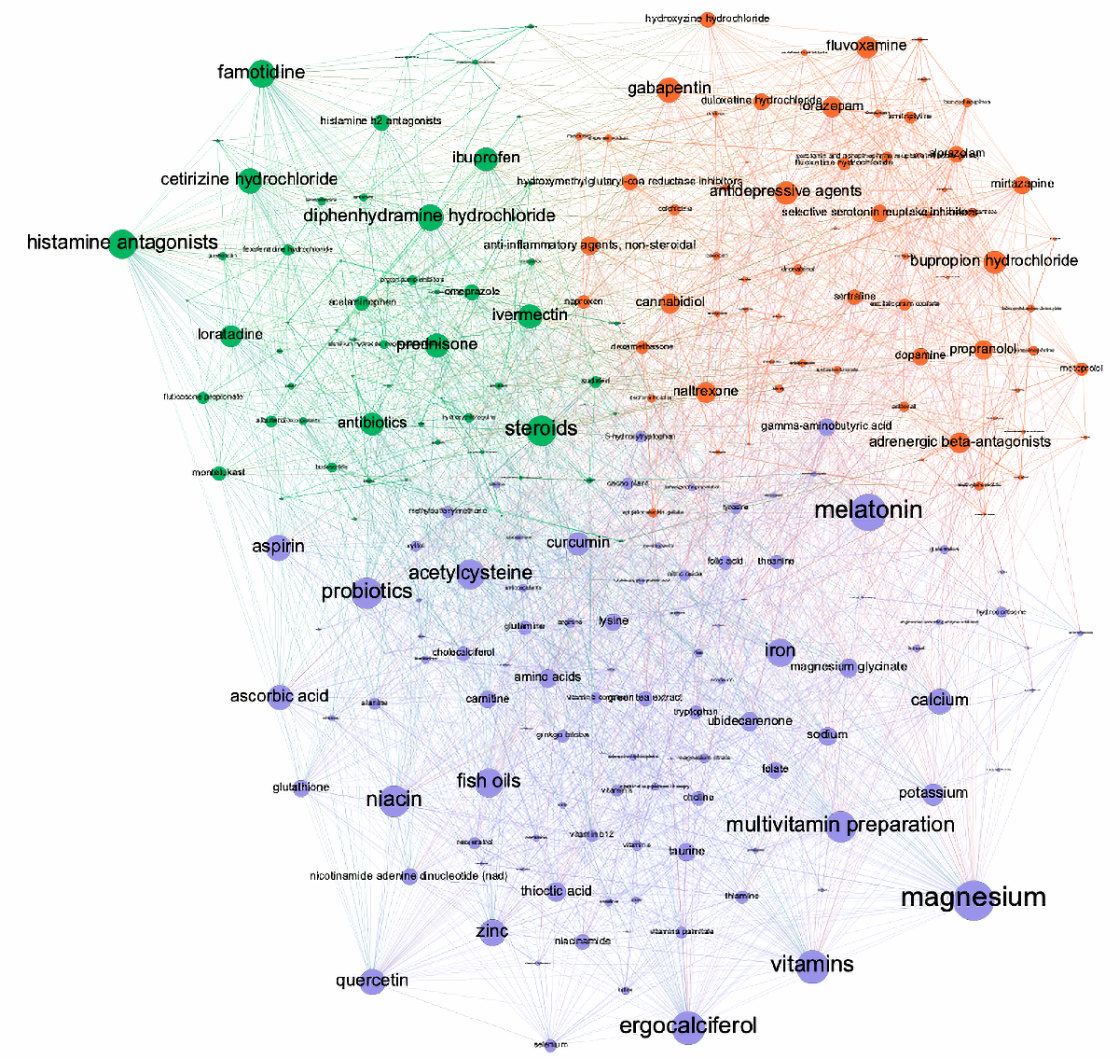
Substance network and clusters. Substances are presented by nodes; the larger the size of a node, the higher degree centrality. Coloring refers to detected communities; violet represents cluster 1, orange refers to cluster 2, and green highlights substances of cluster 3.

Using a resolution of 0.6, three clusters were found. They consisted of 244 nodes and 3570 edges. The modularity value was 0.48, indicating a reasonable partitioning of communities [50,51]. The average clustering coefficient was 0.414. Overall, these scores indicated that the network (Figure 4) had no random structure [47]. Coloring in the network indicates the community and node size degree centrality.

#### Cluster 1

**Figure 5:**
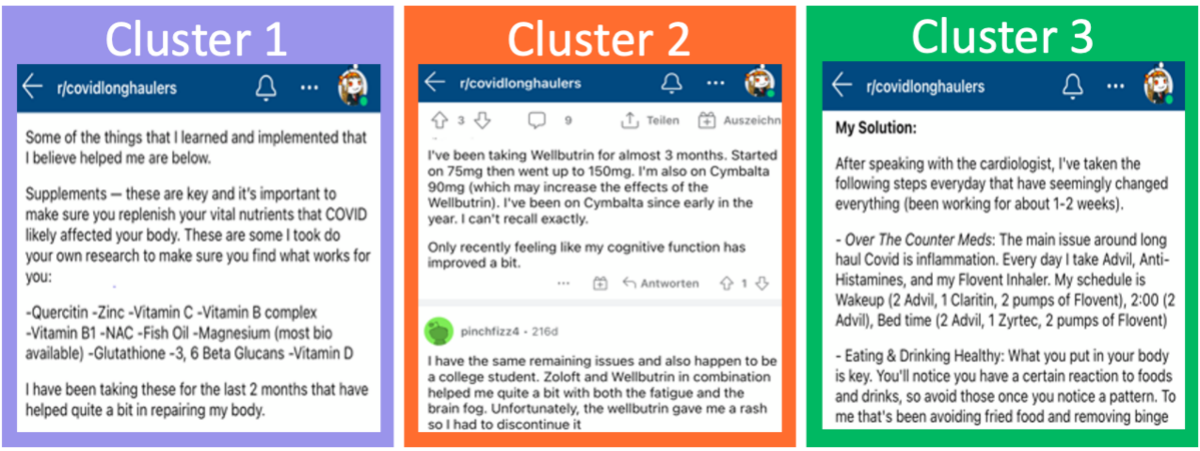
Examples of posts containing typical substance co-occurrences of clusters.

Cluster 1 mainly consisted of supplements and several over-the-counter (OTC) medications, which are often used in the context of flu-like diseases (Figure 5). The top 10 most important substances and the respective substance classes measured by degree centrality are displayed in Table 2. The retrieved entities belong to the drug classes of electrolyte/mineral replacement, vitamins, respiratory tract agents such as acetylsteine, and nutritional supplements such as fish oil or probiotics.

**Table 2:** Characteristics of clusters.

Cluster	Total share of nodes	Ten most important substances (by degree centrality)
1	42.85%	magnesium, melatonin, ergocalciferol, vitamin, multivitamin preparation, niacin, probiotics, acetylsteine, fish oils, zinc
2	29.51%	gabapentin, bupropion hydrochloride, antidepressive agents, fluvoxamine, adrenergic beta-antagonists, naltrexone, lorazepam, cannabidiol, propranolol, nonsteroidal anti-inflammatory agents
3	26.64%	steroids, histamine antagonists, famotidine, diphenhydramine hydrochloride, cetirizine hydrochloride, prednisone, ibuprofen, antibiotics, loratadine, ivermectin

#### Cluster 2

Cluster 2 mostly included prescription medicines such as those used for the treatment of psychological, mental, or neurological disorders (Figure 4). Examples of the 10 most important substances (Table 2) include the anticonvulsant drug gabapentin, antidepressants such as bupropion hydrochloride, and adrenergic β-antagonists such as propranolol. Furthermore, opioid antagonists such as naltrexone, anxiolytics such as lorazepam, and nonsteroidal anti-inflammatory agents such as naproxen incorporated high degree centrality.

#### Cluster 3

Cluster 3 mainly included prescription and OTC medicines that are often used to treat allergic reactions and inflammation (Figure 4). The top 10 important substances (Table 2) belonged to the drug classes of steroids such as prednisone, antihistamines such as famotidine, antibiotics, and nonsteroidal anti-inflammatory agents such as ibuprofen.

## Discussion

### Principal Results

Posts were extracted from an LC-specific subreddit to analyze the discussed substances with the aim of evaluating whether the proposed method could be used to support hypothesis development for DR. In the absence of approved medications for LC (which will also be the case in future pandemic situations), all substances can be considered for off-label use, which makes it difficult to evaluate the potential candidates that apply traditional SMM DR approaches. Instead of filtering substances for off-label use, we considered frequencies and network analysis to facilitate the identification of important substances from the patients’ point of view.

The substances mentioned the most frequently in our feasibility study were antihistamines in general and famotidine, followed by supplements such as magnesium. Moreover, vitamins and steroids were frequently discussed substances. To analyze the strength of the substance-substance combination, the PMI was used to compare the strength of the associations with random associations considering the overall frequency of substances. These substances and their associations formed a nonrandom network consisting of three substance clusters, identified by community detection, implying systematic discussion and usage of substances. For instance, the most frequently mentioned class of substances, histamine antagonists, was found to be highly associated with other inflammatory substances such as steroids.

The clusters, mainly consisting of anti-inflammatory agents and supplements, incorporate the most often mentioned entities. Moreover, medications used for the treatment of psychological, mental, or neurological disorders also formed a cluster. The latter cluster can be assumed to be a less prevalent treatment regime in our sample because its substances occur less frequently. Nevertheless, all clusters reflect the treatment approaches described by the users.

### Supporting DR Hypothesis Generation by Analyzing Patients’ Self-Reports

The results of our feasibility study highlight drugs and self-treatment strategies discussed by long-haulers on Reddit. We were able to successfully identify substance communities (representing different treatment strategies) in the substance network and drugs of high(er) importance to users (based on degree centrality) within these communities. Comparing the results to the current literature, our findings are supported by the successful identification of promising drug candidates already discussed by the scientific community. For instance, according to Crook et al [52], antihistamines are considered potential DR candidates. Significant improvements in long-term symptoms have been reported in case reports [53] and observational studies [54]. This seems to be backed by discussions from long-haulers: antihistamines are the most frequently discussed substances and are important (measured by degree centrality) to the cluster of anti-inflammatory drugs.

Similarly, Crook et al [52] concluded in their review that antidepressants such as serotonin-norepinephrine reuptake inhibitors and selective serotonin reuptake inhibitors could be repurposed for the treatment of LC, as they have been associated with a reduced risk of death or intubation in acute COVID-19 cases [52,55] and a reduction in peripheral inflammatory markers [52,56]. In April 2021, Sukhatme et al [57] conducted review on the mechanisms of action of fluvoxamine and its role in acute COVID-19 treatment. The authors concluded that it “is also tempting to speculate on a role for fluvoxamine in COVID-19 long-haulers.” In April 2022, Khani et al [58] expressed the hypothesis that the majority of LC symptoms might not be directly due to COVID-19 but likely result from COVID-19–associated inflammation and Epstein-Barr virus (EBV) reactivation. The authors argue that fluvoxamine might have beneficial effects in reducing LC symptoms due to the modulatory effects on central mechanisms (eg, reduction of endoplasmic reticulum stress and inflammation). In medication cluster 2, fluvoxamine was the second most important antidepressant, as measured by degree centrality (Table 2). Interestingly, our data are based on posts up to March 2022, and clearly indicate that users already consider this drug to be important in their LC treatment strategies while the scientific community is still working on hypothesis generation.

The other antidepressant that is most frequently mentioned and most central to long-hauler discussions was bupropion hydrochloride, representing a norepinephrine/dopamine reuptake inhibitor (NDRI) [59]. This drug is currently barely discussed in the research community. There seems to be a significant discrepancy in the perception of importance of NDRIs of long-haulers and the scientific community.

Additional drugs that appear to be important to long-haulers but that are barely discussed in clinical research include naltrexone, adrenergic β-antagonists, and prednisone. For instance, prednisone, as a corticosteroid, emerged as the most central steroid used for self-treatment by long-haulers in our study. Chen et al [60] found a high incidence of EBV coinfection in acute COVID-19 cases and concluded that patients may be advised to use a corticosteroid. Following the hypothesis of Khani et al [58] that EBV is also of central importance in LC, corticosteroid could be useful in the treatment of LC, and thus explain the identified central position in the long-haulers discussion identified in this study. In fact, Goel et al [61] reported that systemic steroids are helpful in hastening the recovery of a selected subset of patients with LC [61]. Another recently published single-center interventional pre-post study demonstrated that low-dose naltrexone is safe in patients with LC and may improve well-being (eg, reduce symptomatology) [62].

In summary, reports on clinical research indicate that our proposed method might support the early identification of promising DR candidates. When incorporating patients’ experiences regarding specific drugs, network analysis has proven to be especially useful to slice the data in a meaningful way. In particular, network analysis enables the identification of different (self-) treatment strategies and corresponding drugs beyond raw frequencies. For example, medications used for the treatment of psychological, mental, or neurological disorders represent a medication cluster; however, these drugs are generally reported at lower frequency. This might result from the fact that most of these drugs are only available on prescription and therefore access is limited in contrast to OTCs. Substance community detection and degree centrality highlight the strategies and drugs that otherwise could be overlooked. Furthermore, network analysis allows to distinguish between systematic and random discussions of substances. This is important information, as a systematic discussion might be an indicator for data quality and the knowledge of the crowd. Our results imply that patients’ experiences shared on social media influence others’ self-treatment decisions [63]. Positive experiences reported by users will lead to other users adopting the same approaches, leading to the increase of discussion of potentially helpful substances [64].

However, it is beyond the scope of this study to review all of the identified compounds for their potential relevance to LC DR. We encourage professionals to consider the findings as a starting point for hypothesis generation by narrowing down potential DR candidates to drugs that are important for long-haulers. Clearly, we cannot derive any conclusions about effectiveness based on this analysis. Nevertheless, these drugs appear to be frequently used by long-haulers as treatments. Therefore, these drugs should be further evaluated by the scientific community to determine whether they might be effective or even harmful, which would also be of relevance to communicate from a public health perspective.

### Limitations and Future Work

Our study has several limitations in line with comparable studies based on similar methods. As our NER algorithm relies on pretrained models, the error analysis we performed implies that a custom model trained on annotated examples of the posts used in this study would increase the accuracy of the results. To avoid missing entities in the normalization process (eg, due to the use of slang), a customized dictionary could be defined, which links slang terms of substances used by patients to their medical terminology. Further, substances that fail in the normalization process could be revised and normalized manually. However, the performance of the NER algorithm can be considered to be appropriate. Other studies applying pretrained NER algorithms to extract medical entities from Reddit data showed similar F1 scores. Foufi et al [49] used PKDE4J [65] to identify biomedical substances from disease-specific subreddits, and 71.48% of the extracted entities were correctly labeled. Šćepanović et al [66] evaluated six state-of-the-art pretrained language models in a bidirectional long short-term memory–conditional random field (BiLSTM-CRF) model to identify medical entities in disease-specific subreddits; the F1 scores ranged from 0.64 to 0.73. Approaches combining different pretrained language models can improve performance. For example, Šćepanović et al [66] built a customized NER system by combining a BiLSTM-CRF sequence labeling architecture with contextual embeddings, which scored 0.71 on symptoms and 0.77 on drugs*.*

### Determining the Outcome of Substances

We analyzed the importance of substances in the long-haulers’ discussions, but we did not analyze whether the substances were helpful in terms of the outcomes, which should be considered in future studies. Several approaches can be applied to approximate outcomes. The gold standard is the manual examination of medication-specific posts by medical professionals incorporating domain knowledge. Further programmatic approaches could include analyses of average sentiments regarding drugs to determine whether a treatment is perceived to be useful by users [67]. Another possibility could be to capture correlations between substances and effects mentioned in a sentence by the application of dependency parsing [68] or by implementing an observational study design [69]. However, reliable assessment of causality of underlying treatment effects is impossible because of various limitations [19]; for instance, inaccurate use of medical terminology by users would bias the results, even if machine-learning algorithms perform with perfect accuracy. Moreover, our analysis indicates a trend of polymedication, which would confound the analysis of single substances. Furthermore, the data quality is low compared with data obtained in traditional study designs, and several confounders such as demographic variables are unknown. In general, even if holistic patient information is available, social media data should be interpreted with caution. For instance, ivermectin was considered a potential DR candidate for acute COVID-19. However, this is suspected to be supported by flawed research [70] and it was later found to be ineffective in randomized clinical trials [71]. While we cannot evaluate the efficacy of ivermectin in patients with LC, it can be assumed that users’ treatment decisions may also be influenced by potentially flawed information.

### Generalizability of Results and Inference of Indications

Even though important substances were found from the users’ perspective, the results cannot be generalized for all patients with LC since demographics or symptom distributions were not analyzed. Although previous studies [26,27] indicate that the spectrum of symptoms in the subreddit broadly agrees with recently published studies on LC [26,27], it can be assumed that the treatment choice of users depends on their symptoms. Therefore, the implementation of a bimodal network to reveal correlations between symptoms and medications could be useful. Moreover, the demographic distribution of users might not be representative of the entire long-hauler population. In fact, research suggests that the distribution of Reddit is skewed by age and gender [72], which is a major limitation to SMM in general [19], indicating that the data source represents a young male subpopulation [72]. There are two possible improvements in future SMM studies: (1) combining data from multiple platforms could lower user bias depending on the specific platform; and (2) algorithms can be applied to infer demographic variables and analyze diverse user data, including metadata [19].

### Conclusions

In this feasibility study, we tested the application of SMM methods to support the development of hypotheses on LC DR. To this end, we extracted substance entities to analyze frequencies and co-occurrences and subsequently used them to identify substance clusters. Our results highlight certain approaches to DR, such as antihistamines, steroids, or antidepressants, while also indicating that patients experiment with a wide range of substances in a systematic manner. This feasibility study demonstrates that network analysis can be used to characterize the medication strategies discussed. Comparison with existing literature indicates that the approach identifies substances that are plausible candidates for drug repurposing. The comparison also showed that some substances are important from the users’ point of view while they are barely discussed in the scientific community. These substances should be reviewed by experts to assess their potential efficacy or harmfulness. The result could either lead to a DR hypothesis or underline the need to communicate the potential risks of the drug in the community of long-haulers. In the context of a pandemic, the proposed method might be used to support DR hypothesis development by prioritizing substances that are important to users in a cost- and time-effective manner.

## Appendix

Multimedia Appendix 1 Link flair text (LFT) tags included and excluded from the analysis, and total substances extracted from posts.

## Abbreviations

BiLSTM-CRF: bidirectional long short-term memory–conditional random field
DR: drug repurposing
EBV: Epstein-Barr virus
LC: long COVID
NDRI: norepinephrine/dopamine reuptake inhibitor
NER: named-entity recognition
OTC: over the counter
PMI: pointwise mutual information
SMM: social media mining
UMLS: unified medical language system
