# Supplementary material for: Social Media Mining of Long-COVID Self-Medication Reported by Reddit Users: Feasibility Study to Support Drug Repurposing
Source: JMIR Form Res. 2022 Oct 3;6(10):e39582. doi: 10.2196/39582 (PMC9531770; doi:10.2196/39582)
Supplement: Multimedia Appendix 1 [file formative_v6i10e39582_app1.docx]

**Multimedia Appendix 1. Link flair text (LFT) tags included and excluded from the analysis, and total substances extracted from posts.**

| **LFT Tags** | **Included in analysis** |
| --- | --- |
| Symptoms | yes |
| Personal Story | yes |
| Question | yes |
| Symptom relief/advice | yes |
| TRIGGER WARNING | yes |
| Update | yes |
| Vent/Rant | yes |
| Mental Health/Support | yes |
| Recovery/Remission | yes |
| Improvement | yes |
| Research | no |
| Humor | no |
| Commorbidities | yes |
| Vaccine | no |
| Reinfected | yes |
| Article | no |
| Omicron | yes |
| Post-vaccine | no |
| 'Family/Friend Support' | yes |
| video | no |
| Announcement | no |
| Mod! | no |
| Treatment | yes |
| Meta | no |
| 'Gastrointestinal issues' | yes |
| Symptoms | yes |
| Test results | no |
| Mental Health | yes |

| **Substance** | **Number of posts mentioning the substance** |
| --- | --- |
| histamine antagonists | 850 |
| famotidine | 801 |
| magnesium | 725 |
| vitamins | 607 |
| steroids | 598 |
| melatonin | 552 |
| aspirin | 550 |
| niacin | 531 |
| ergocalciferol | 514 |
| adrenergic beta-antagonists | 460 |
| cetirizine hydrochloride | 449 |
| ivermectin | 360 |
| antibiotics | 325 |
| prednisone | 304 |
| iron | 290 |
| antidepressive agents | 286 |
| quercetin | 285 |
| mirtazapine | 279 |
| ibuprofen | 267 |
| calcium | 267 |
| acetylcysteine | 265 |
| potassium | 256 |
| selective serotonin reuptake inhibitors | 245 |
| loratadine | 244 |
| zinc | 239 |
| probiotics | 237 |
| ascorbic acid | 227 |
| bupropion hydrochloride | 223 |
| fluvoxamine | 220 |
| diphenhydramine hydrochloride | 208 |
| propranolol | 172 |
| gabapentin | 168 |
| hydroxymethylglutaryl-coa reductase inhibitors | 165 |
| multivitamin preparation | 152 |
| metoprolol | 139 |
| hydrocortisone | 131 |
| cannabidiol | 130 |
| montelukast sodium | 129 |
| nicotinamide adenine dinucleotide (nad) | 129 |
| acetaminophen | 127 |
| non-steroidal anti-inflammatory agents | 122 |
| lorazepam | 115 |
| fish oils | 112 |
| albuterol | 107 |
| hydroxyzine hydrochloride | 105 |
| omeprazole | 105 |
| fluoxetine hydrochloride | 103 |
| curcumin | 102 |
| sodium | 96 |
| testosterone | 96 |
| fluticasone propionate | 93 |
| thiamine | 93 |
| sertraline hydrochloride | 87 |
| naltrexone | 87 |
| duloxetine hydrochloride | 87 |
| alprazolam | 84 |
| thioctic acid | 81 |
| taurine | 79 |
| histamine h2 antagonists | 78 |
| escitalopram oxalate | 74 |
| nicotine | 73 |
| ubidecarenone | 72 |
| dronabinol | 70 |
| marihuana | 69 |
| adderall | 65 |
| niacinamide | 63 |
| glutathione | 63 |
| magnesium glycinate | 60 |
| proton pump inhibitors | 60 |
| maraviroc | 60 |
| ginkgo biloba | 56 |
| vaccines | 56 |
| selenium | 56 |
| guaifenesin | 55 |
| gamma-aminobutyric acid | 54 |
| green tea (dietary) | 53 |
| colchicine | 51 |
| amitriptyline | 51 |
| choline | 51 |
| vitamin b12 | 51 |
| theanine | 49 |
| anticoagulants | 48 |
| lysine | 46 |
| serotonin and norepinephrine reuptake inhibitors (SNRIs) | 46 |
| carnitine | 45 |
| amino acids | 45 |
| zolpidem tartrate | 42 |
| antacids | 41 |
| dexamethasone | 39 |
| naproxen | 39 |
| montelukast | 38 |
| vitamin b complex | 38 |
| aldosterone | 38 |
| fexofenadine hydrochloride | 37 |
| adrenal cortex hormones | 37 |
| modafinil | 36 |
| trazodone | 36 |
| psilocybin | 35 |
| glutamine | 35 |
| clonazepam | 35 |
| cholecalciferol | 34 |
| benzodiazepines | 33 |
| ozone | 33 |
| biotin | 33 |
| methylphenidate hydrochloride | 32 |
| alanine | 32 |
| ketamine | 31 |
| ivabradine | 31 |
| pregabalin | 31 |
| ondansetron hydrochloride | 29 |
| folate | 29 |
| lisdexamfetamine dimesylate | 28 |
| meclizine | 28 |
| meloxicam | 27 |
| iodine | 27 |
| valacyclovir hydrochloride | 27 |
| duloxetine | 27 |
| arginine | 27 |
| resveratrol | 27 |
| catecholamines | 27 |
| sertraline | 26 |
| tyrosine | 26 |
| angiotensin-converting enzyme inhibitors | 26 |
| sudafed | 26 |
| lamotrigine | 26 |
| tums | 26 |
| tryptophan | 25 |
| quetiapine fumarate | 25 |
| magnesium citrate | 25 |
| vitamin d supplement therapy | 25 |
| hydroxychloroquine | 25 |
| lisinopril | 25 |
| pyridostigmine bromide | 24 |
| betaine | 24 |
| ashwagandha preparation | 24 |
| atenolol | 23 |
| nattokinase | 23 |
| 5-hydroxytryptophan | 23 |
| aripiprazole | 23 |
| norepinephrine | 22 |
| folic acid | 22 |
| fluconazole | 22 |
| xylitol | 22 |
| acetylcholine | 22 |
| progesterone | 22 |
| valerian | 22 |
| ketotifen | 22 |
| amantadine | 22 |
| vitamin k | 21 |
| afrin | 21 |
| diclofenac | 21 |
| pravastatin | 21 |
| vitamin a palmitate | 20 |
| nortriptyline | 20 |
| naproxen sodium | 20 |
| midodrine | 20 |
| fexofenadine | 20 |
| diazepam | 20 |
| fluvoxamine maleate | 20 |
| creatine | 20 |
| pantoprazole | 19 |
| budesonide | 19 |
| topiramate | 19 |
| vitamin e | 19 |
| betaine hydrochloride | 18 |
| cortisone | 18 |
| analgesics | 18 |
| barium | 18 |
| valganciclovir hydrochloride | 18 |
| sulfur | 17 |
| adenosine triphosphate | 17 |
| bisoprolol | 17 |
| fludrocortisone | 17 |
| citalopram | 17 |
| dramamine | 17 |
| doxylamine | 17 |
| diphenhydramine | 17 |
| celecoxib | 17 |
| levocetirizine dihydrochloride | 17 |
| desloratadine | 16 |
| decongestants | 16 |
| agmatine | 16 |
| hallucinogens | 16 |
| riboflavin | 15 |
| ciprofloxacin | 15 |
| methylsulfonylmethane | 15 |
| central nervous system stimulants | 15 |
| excedrin | 14 |
| nootropic agents | 14 |
| menthol | 14 |
| methylphenidate | 14 |
| quarantine | 14 |
| pyridoxine | 14 |
| creatinine | 14 |
| advair | 14 |
| nitrates | 14 |
| budesonide | 14 |
| acetazolamide | 14 |
| monolaurin | 13 |
| carnosine | 13 |
| amoxicillin | 13 |
| mentha piperita | 13 |
| brivudine | 13 |
| metformin | 13 |
| paroxetine hydrochloride | 13 |
| lactoferrin | 13 |
| methylprednisolone sodium succinate | 13 |
| dextromethorphan | 13 |
| venlafaxine hydrochloride | 13 |
| glucosamine | 12 |
| doxy | 12 |
| lanthanum | 12 |
| threonate | 12 |
| levocetirizine | 12 |
| epigallocatechin gallate | 12 |
| green tea extract | 12 |
| lysergic acid diethylamide | 12 |
| nyquil | 12 |
| azithromycin | 12 |
| midomafetamine | 12 |
| chamomile tea | 12 |
| promethazine hydrochloride | 11 |
| serratiopeptidase | 11 |
| benphothiamine | 11 |
| eszopiclone | 11 |
| citrulline | 11 |
| acyclovir | 11 |
| pepsin a | 10 |
| spironolactone | 10 |
| flax | 10 |
| charcoal | 10 |
| huperzine a | 10 |
| endorphins | 10 |
| listerine | 10 |
| hydroxychloroquine sulfate | 10 |
| doxycycline | 10 |
| lidocaine | 10 |
| kombucha tea | 10 |
| metoclopramide hydrochloride | 10 |
| leronlimab | 10 |
| magnesium threonate | 10 |
